# Transcatheter Heart Valve Implantation in Bicuspid Patients with Self-Expanding Device

**DOI:** 10.3390/bioengineering8070091

**Published:** 2021-07-01

**Authors:** Salvatore Pasta, Stefano Cannata, Giovanni Gentile, Valentina Agnese, Giuseppe Maria Raffa, Michele Pilato, Caterina Gandolfo

**Affiliations:** 1 Department of Engineering, University of Palermo, 90128 Palermo, Italy; 2 Department for the Treatment and Study of Cardiothoracic Diseases and Cardiothoracic Transplantation, IRCCS-ISMETT, 90127 Palermo, Italy; stcannata@ismett.edu (S.C.); vagnese@ismett.edu (V.A.); graffa@ismett.edu (G.M.R.); mpilato@ismett.edu (M.P.); cgandolfo@ismett.edu (C.G.); 3 Department of Diagnostic and Therapeutic Services, IRCCS-ISMETT, 90127 Palermo, Italy; gigentile@ismett.edu

**Keywords:** transcatheter aortic valve implantation, bicuspid aortic valve, finite-element analysis, fluid–solid interaction

## Abstract

Bicuspid aortic valve (BAV) patients are conventionally not treated by transcathether aortic valve implantation (TAVI) because of anatomic constraint with unfavorable outcome. Patient-specific numerical simulation of TAVI in BAV may predict important clinical insights to assess the conformability of the transcathether heart valves (THV) implanted on the aortic root of members of this challenging patient population. We aimed to develop a computational approach and virtually simulate TAVI in a group of n.6 stenotic BAV patients using the self-expanding Evolut Pro THV. Specifically, the structural mechanics were evaluated by a finite-element model to estimate the deformed THV configuration in the oval bicuspid anatomy. Then, a fluid–solid interaction analysis based on the smoothed-particle hydrodynamics (SPH) technique was adopted to quantify the blood-flow patterns as well as the regions at high risk of paravalvular leakage (PVL). Simulations demonstrated a slight asymmetric and elliptical expansion of the THV stent frame in the BAV anatomy. The contact pressure between the luminal aortic root surface and the THV stent frame was determined to quantify the device anchoring force at the level of the aortic annulus and mid-ascending aorta. At late diastole, PVL was found in the gap between the aortic wall and THV stent frame. Though the modeling framework was not validated by clinical data, this study could be considered a further step towards the use of numerical simulations for the assessment of TAVI in BAV, aiming at understanding patients not suitable for device implantation on an anatomic basis.

## 1. Introduction

Bicuspid aortic valve (BAV) occurs in 1–2% of the worldwide population and represents the most common congenital cardiac abnormality, causing the highest morbidity and mortality among other cardiac defects [1]. Individuals with BAV have high risk of developing valvular diseases, including a dilated aorta at birth and rapid valve leaflet degeneration as characterized by calcification. Definitely, BAVs are more prone to developing valve stenosis due to increased mechanical stimuli of hemodynamic origin and having a predisposition to calcium formation [2]. This stenotic condition typically manifests at a younger age than the stenosis of individuals with the morphologically normal tricuspid aortic valve. The standard clinical approach for treating stenotic BAV is surgical replacement with native tissue or a bioprosthetic heart valve.

As transcathether aortic valve implantation (TAVI) has become effective in the clinical management of stenotic patients, there is a growing interest in expanding this approach in younger and low-risk patients. However, the majority of these patients have congenital BAV at the time of clinical observation so that BAVs are currently excluded from all trials of TAVI performance in humans. Recent studies documented the clinical feasibility of TAVI in BAV as shown by the comparable degree of paravalvular leakage (PVL) between bicuspid and tricuspid patients when the leak was graded equal or greater than mild [3,4,5]. No significant differences in the device success rate, risk of annulus rupture and valve migration of BAV patients versus tricuspid patients were found. However, efforts should be made to optimize transcatheter heart valve (THV) sizing and positioning to reduce PVL and conduction abnormalities. As TAVI in BAV has been believed safe because of the new generation of THVs, clinicians should be aware of inherent technological limitations of current bioprosthesis when performing “off-label” applications in low-risk profile patients. For these reasons, patient-specific computer simulations could be an attractive solution to facing challenges with TAVI in bicuspid patients.

This study sought to determine the biomechanical implication of TAVI in severe stenotic BAV by developing a computational framework using finite-element analyses for the THV deployment and smoothed particle hemodynamic (SPH) technique for flow assessment. Numerical simulations were performed on n.6 patients with stenotic BAV to determine (i) the deformed configuration of THVs, (ii) the contact pressure between the aortic wall and the THV stent frame and (iii) the flow velocity map and regions at risk of PVL. Findings on the performance of TAVI in BAV are discussed.

## 2. Materials and Methods

### 2.1. TAVI Procedure and Bicuspid Classification

This study included n.6 patients with stenotic BAV treated with TAVI as dictated by the heart team and the clinical risk profile of each patient. All patients received the Medtronic Evolut Pro (Medtronic Inc., Grand Rapids, MI, USA) using device diameters ranging from 23 mm to 29 mm. The optimal device size was based on measurements of the annulus dimension and size collected from pre-procedural CT imaging. The latter was performed using a 64-row CT scanner (GE Healthcare, Westborough, MA, USA) with z-resolution of 0.625 mm and gantry rotation of 320 ms. The BAV phenotype was classified by an experienced radiologist according to the number of cusps; presence of raphes; and spatial position and symmetry of raphes and cusps. The Sievers classification scheme was used to group BAV in pure BAV and left-right cusp fusion [6]. For all patients, BAVs were congenital abnormalities as determined by echocardiographic assessment at in-hospital admission. Table 1 summarizes clinical demographic data, BAV phenotype and baseline CT measurements for each patient. Our local ethics review committees approved the study, and patients gave informed consent to their inclusion in the study.

### 2.2. Computational Analysis

Simulations consisted of (a) patient-specific reconstructions of both the aortic root and calcification; (b) parametric modeling of native bicuspid leaflets; (c) simulation of the pre-TAVI configuration; (d) crimping and deployment of THV in the patient model; (e) fluid–solid interaction (FSI) analysis for simulating prosthetic valve dynamics and assessing region of PVL.

### 2.3. Anatomic Models

Pre-TAVI CT images at diastolic phase were processed using Mimics (Materialise, Leuven, Belgium) to segment the aortic root anatomy and calcification using different grey values and multiple masks [7,8,9,10]. This was accomplished by first applying semi-automatic region growing and then finalizing the anatomic mask by manual editing. Calcific plaques were extracted in terms of both spatial location and dimension. Native bicuspid leaflet anatomies were not clearly visible at CT scan so that a parametric modeling approach based on anatomic measurements was adopted to model the stenotic BAV. Specifically, several spline curves were used to model the free edge of the BAV leaflet and the cusp-to-commissure attachment with the aortic wall. Using these bounding curves, native BAV leaflets were modeled by a multi-patch network of NURBS surfaces as previously conducted by our group [7]. Using ICEM meshing software (Ansys v.18, ANSYS, Inc., Canonsburg, PA, USA), both the aortic root and native BAV leaflets were discretized with structured quadrilateral shell elements (S4). Calcific plaques were meshed by a combination of hexahedral and tetrahedral solid elements (element size of 0.1 mm).

### 2.4. THV Model

The Evolut Pro (Medtronic, Fridley, MI, USA) THV is characterized by a supra-annular structure with a self-expanding nitinol frame and porcine pericardial tissue leaflets. The device also includes an outer sealing skirt to prevent PVL. The CAD model of the Evolut Pro was generated combining geometrical measurements collected from a high-resolution micro-CT scanner (Skyscan 1272, Bruker, Bruker, MA, USA) with reverse engineering of the metallic frame. Then, the THV model was meshed with 315,653 structured-hexahedral solid elements with reduced integration and hourglass control. Both the sealing skirt and the prosthetic valve leaflets were not included during TAVI simulation but were modeled after the stent frame deployment by mapping their geometries onto the implanted stent frame at initial stress-free closed configuration [11]. The 23 mm and 29 mm THVs were obtained as scaled versions of the 26 mm frame geometry.

### 2.5. Material Models

The biomechanical behavior of the aortic root and native BAV leaflets was assumed to be hyperelastic with isotropic materials using a two-term Yeoh constitutive relation and density ρ = 1060 kg/m^3^. Specifically, the material parameters of the aortic root were C1 = 0.015 MPa and C2 = 0.158 MPa while the native BAV leaflets had C1 = 0.008 MPa and C2 = 0.048 MPa [12]. The shell aortic root and native BAV leaflets had uniform thickness of 2.0 and 0.5 mm, respectively. In a different way, a linear elastic model was used to model the stiff calcific plaque (E = 10 MPa and ν = 0.49, ρ = 1060 kg/m^3^) [13]. The stent frame was modeled with NiTi alloy material properties using the built-in model implemented in Abaqus and assuming a superelastic behavior. Material parameters describing the NiTi alloy behavior (n.14 material descriptors) were based on the study proposed by Auricchio et al [11]. Because of the lack of constitutive parameters, the biomechanical behavior of both the skirt and THV valve leaflets was assumed to be linear elastic material (E = 1 MPa and ν = 0.49, ρ = 1060 kg/m^3^) [14].

### 2.6. TAVI Model

Numerical analyses of TAVI procedure were developed in Abaqus/Explicit using a quasi-static approach by monitoring energy and ensuring the ratio of kinetic energy to internal energy remained less than 10%. A semi-automatic mass scaling approach was applied for the entire model while the general contact algorithm was adopted to account for interaction among anatomic parts and THV.

**Figure 1:**
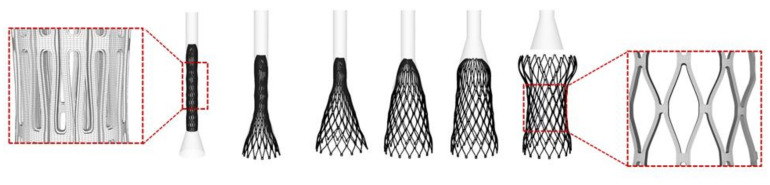
Different steps of the kinematics of THV deployment by the sleeve lifting from the initial crimped configuration.

To generate enough space for THV deployment, a pre-TAVI configuration was obtained applying a pressure differential waveform (i.e., a pressure difference between the ventricle and the aorta) on the closed native BAV leaflet surface. The device was crimped using a cylindrical surface gradually moved along the radial direction from the initial device diameter to the final diameter of 6 mm using frictionless contact conditions. The crimper was meshed using quadrilateral surface elements with ρ = 1060 kg/m^3^. In a second and separate simulation, the crimped THV model was imported in the patient anatomy with its residual stresses from the previous crimping simulation, together with the geometry of the sleeve. Both the stent and sleeve were positioned in the aortic root of each patient, with the proximal end of the THV stent located at 6 mm from the annulus as recommended by the manufacturer. By pulling the sleeve towards the distal ascending aorta and releasing the stent, because of its residual stresses, the THV stent was gradually deployed inside the patient aortic root anatomy (Figure 1). The pull out of the rigid sleeve was performed by a uniform displacement of 30 mm. Longitudinal and circumferential displacements of the aortic root were fixed. A video of the stent deployment is provided in the Supplementary Materials.

### 2.7. SPH Modeling

**Figure 2:**
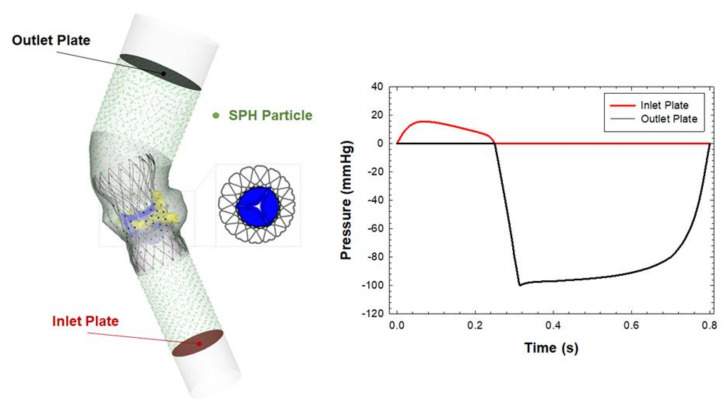
Model adopted for the fluid–solid interaction analysis and physiological pressure boundary conditions adopted to move the SPH particle.

The SPH method is advantageous as compared to coupled Eulerian–Lagrangian techniques and can be ideal for simulating fluid dynamic phenomena with complex interactions between solid and fluid parts. The SPH is a meshless numerical method defining a body by a point collection, instead of using nodes and elements. In this study, the blood was assumed to be Newtonian fluid with ρ = 1060 kg/m^3^ and viscosity of 0.0035 Pa using the pressure–density relation governed by the linear Hugoniot equation of state (artificial sound speed of c0 = 145 m/s). Particle distribution of the fluid domain had a spatial resolution of 0.5 mm in agreement with mesh sensitivity analysis carried out by Mao et al. [15]. Particle motion was developed by pressure boundary conditions exerted on the blood volume by two rigid plates as shown in Figure 2. Specifically, the pressure gradient between the left ventricle and aorta was generated by means of representative physiological pressure profiles from literature data [16]. The proximal and distal ends of the aortic root were sufficiently extended to ensure a fully developed flow while the beginning of the systole was used as the starting point of the flow simulation. We assumed a cardiac beat of 0.8 s and simulated two beats to reduce transient effects. To allow FSI, contact was enabled between particles and prosthetic valve leaflets but the aortic wall was considered to be a rigid wall. For post-processing, the particle flow data were interpolated on a new hexahedral element mesh so that the flow contour map is shown instead of the particle point collection.

## 3. Results

The structural mechanics and hemodynamic of TAVI simulation on stenotic bicuspid patients are shown in terms of (i) deformed configuration of Evolut Pro device, (ii) contact pressure on the inner aortic root wall induced by the interaction of the stent with the aortic wall and (iii) flow contour maps during cardiac beating at different heights of the implanted device.

**Figure 3:**
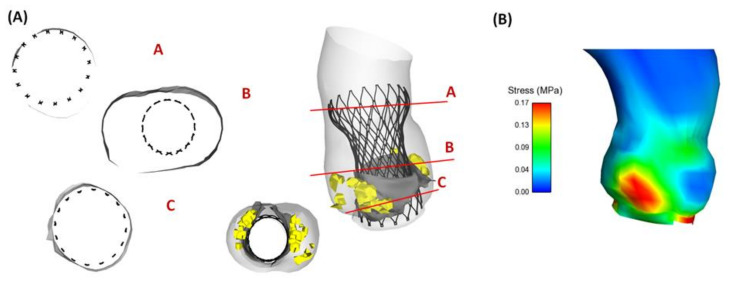
(**A**) Deformed configuration of deployed THV showing the contact between the aortic wall and THV at different cross-section and (**B**) resulting maximum principal stress distribution for Patient #5 with a pure BAV phenotype.

Figure 3 shows the deployed THV and the resulting maximum principal stress distribution for a representative patient with a pure phenotype of stenotic BAV leaflets (Patient #5). The numerical model exhibited a good positioning of the THV stent frame to the aortic root wall, resembling a uniform contact at aortic annulus. The ascending aorta of Patient #5 had a slightly dilated aorta with diameter of 38.5 mm, and this led to less contact pressure magnitude between the THV and ascending aorta. Moreover, the map of maximum principal stress was mostly characterized by local maxima in the contact area of the aortic root with either the THV stent frame or the calcific plaques.

**Figure 4:**
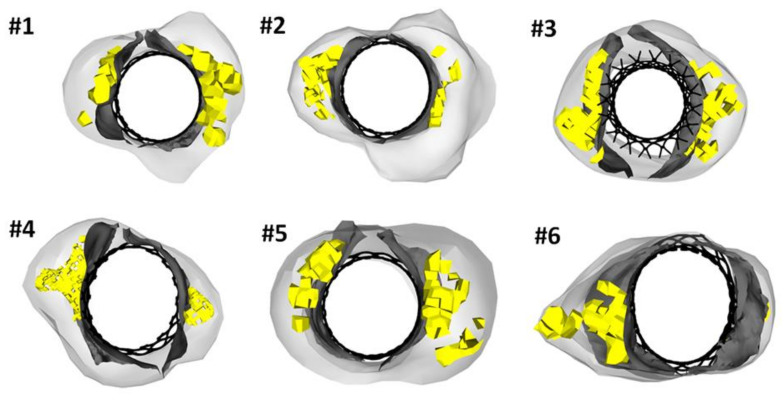
Deformed configuration of TAVI for each patient at a cross-section corresponding to the sinus of Valsalva.

Figure 4 highlights the deformed configurations of implanted THVs for each stenotic bicuspid patient. We observed that the THV stent frame was characterized by a slightly elliptical shape at aortic annulus to accommodate the oval bicuspid anatomy in Patient #4 and #6. In other patient cases, the stent frame had a circular shape at the implanted configuration. Indeed, the degree of THV deformity is highly variable from patient to patient, with relevant frame distortions caused by the amount and position of calcifications with respect to the aortic root wall. It can be clearly observed that Patient #6 with right-left fusion bicuspid phenotype had remarkable distortion of the THV frame at the sinus of Valsalva. In a different way, the large annulus size of Patient #3 led to a more circular configuration of the deployed THV. This was likely caused by the large dimension of the aortic valve annulus of Patient #3 so that the device was implanted in the proximal left ventricular outflow tract (implantation depth of 12.5 mm).

**Figure 5:**
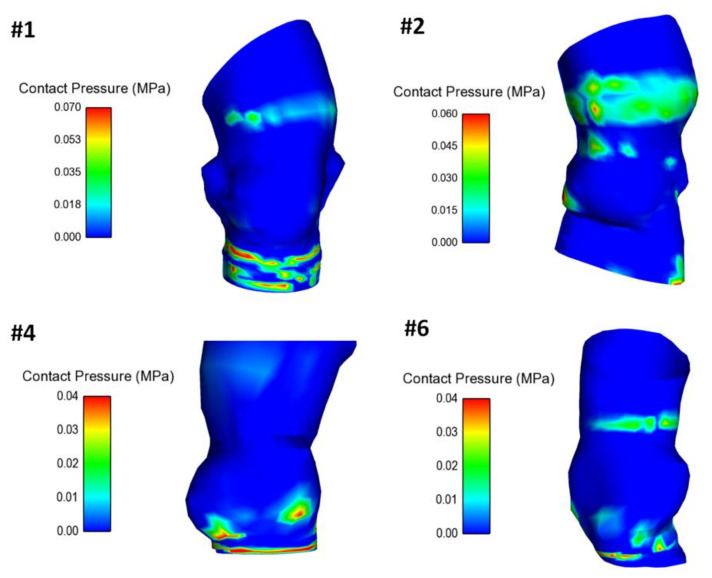
Distribution of the contact pressure between the inner aortic wall surface and the deployed THV for four patients.

The map of contact pressure was investigated in the inner surface of the aorta root wall to analyze the interaction between the self-expandable THV and vessel luminal wall (Figure 5). Indeed, the self-expandable THV remained anchored to the aortic wall because of the radial force that the superelastic stent frame exerts on the inner and more compliant vessel wall. The area at the highest contact pressure corresponded well with the aortic annulus and the ascending aorta thanks to the anchoring structure of the supra-annular region of the THV.

**Figure 6:**
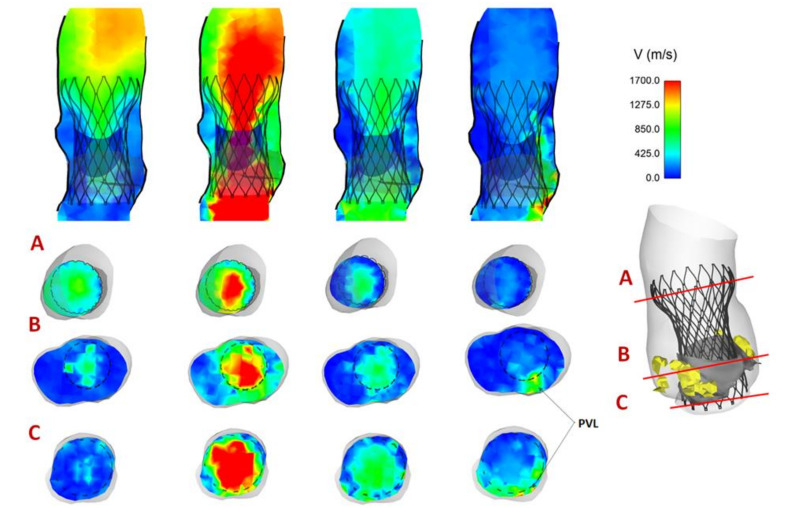
Map of flow velocity for Patient #5 showing the region of PVL; flow velocity shown from acceleration, to peak systole, early diastole, ending with late diastole, at three analysis planes.

**Figure 7:**
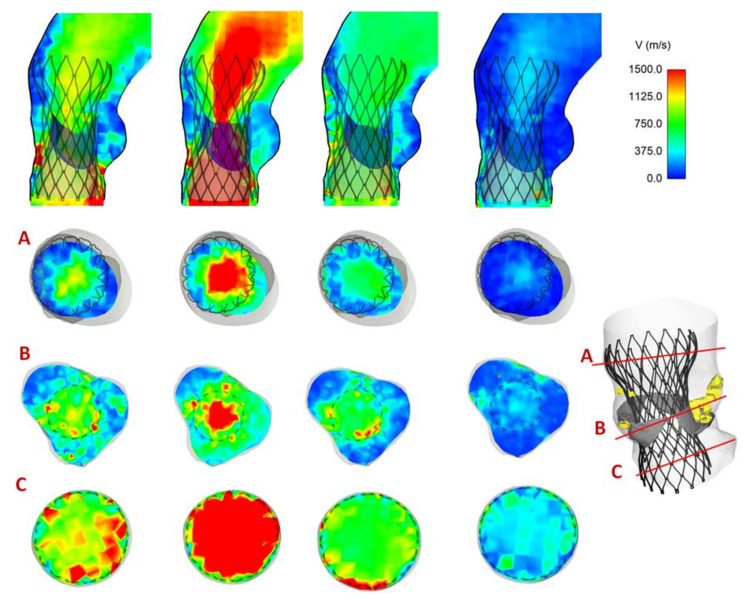
Map of flow velocity for Patient #2 with no sign of PVL; flow velocity shown from acceleration, to peak systole, early diastole, ending with late diastole, at three analysis planes.

Maps of flow velocity were assessed at cross-section of the vessel and at different analysis planes, including the aortic annulus inflow, mid-height and outflow from the device. Figure 6 shows the flow velocity maps for Patient #5 with a trivial presence of PVL. Specifically, the velocity contour map of Patient #5 in different planes shows a mean central flow jet during acceleration and peak systolic phase when prosthetic valve leaflets are opened. Although the THV leaflets are closed at late diastole, two minor regions of PVL can be observed near the commissures of native BAV leaflets. In a different way, Figure 7 illustrates the blood flow map for Patient #2 with no presence of PVL at diastole.

## 4. Discussion

A computational framework for assessing the structural mechanics and hemodynamic performance of THV in stenotic bicuspid patients was here developed. Bicuspid patients do not undergo TAVI procedures because of anatomical constraints not commonly observed in the tricuspid patient population. This makes outcomes of TAVI in bicuspid patients particularly challenging, so computational modeling may help to assess the feasibility of TAVI in such a patient population. In a few patients, the THV stent frame appeared to deform asymmetrically in the aortic root as characterized by a slight elliptical device configuration. Changes in the frame distortion occurred differently from patient to patient according to the amount and spatial position of stiff calcific plaques. The contact pressure between the stent frame and the aortic wall was evaluated to quantify the anchoring forces, and this was found to be highest at aortic annulus and supra-annular structure of the Evolut Pro device. The FSI approach using the SPH methodology to reduce the complexity of the interaction between fluid and solid parts allowed visualizing the flow patterns over the cardiac cycle and the regions at risk of PVL. Although findings were not verified by clinical data based on post-TAVI CT and echocardiography, this computational approach allowed predicting several clinically relevant insights on TAVI in patients with BAV.

TAVI represents a promising alternative strategy to open-chest surgery in patients with a stenotic aortic valve and contraindications due to advanced age. The BAV anatomy is characterized by a large left ventricular outflow tract and aortic annulus and ascending aortic ectasia at birth. In this way, clinical trials for the assessment of THV efficacy and safety in humans did not include bicuspid patients because of the likelihood of an oval expansion and underperforming long-term clinical outcome. Recently, TAVI in stenotic BAVs [3,4,5] was found to be clinically feasible as this was determined by device improvements and increased operator experience. Early findings of the safety of TAVI in BAVs were achieved with the Edwards SAPIEN 3 Ultra using a balloon for the device deployment and positioning [17]. It is proposed that the good performance of SAPIEN 3 in BAVs is likely due to the high radial strength offered by the metallic steel frame. Recently, clinical findings with the Evolut R and Pro devices on bicuspid patients have been documented [18]. It is worth noting that both the self-expanding and balloon-expandable devices are characterized by a longer sealing skirt to avoid the leakage as compared to the previous generation. However, the lack of large multicenter studies and the presence of complications such as PVL and conduction abnormalities are still determining challenges in the application of TAVI in bicuspid patients. As TAVI begins to move into younger and lower-risk patient profiles, clinicians should be aware of inherent technology limitations of THVs and stratify borderline patients using innovative approaches such as computer modeling.

Although several studies evinced the efficacy of simulations for planning TAVI procedures, there is limited literature concerning the in-silico modeling in BAVs. For the tricuspid aortic valve, patient-specific simulations of TAVI demonstrated the presence of aortic wall stress concentration [19], the impact of deployment strategy [20], calcification patterns [21] and native leaflets [22] with either self-expanding [23] and balloon-expandable THVs [24]. Recently, a combined structural and hemodynamic analysis was performed with the Living Heart Human model [25], which is capable of simulating the cardiac beating of the whole heart. The broad numerical methodology represented a valid and predictive tool not only to determine the performance of THVs but also for improving valve design for reducing the incidence of reported complications. In the setting of bicuspid patients, Lavon et al. [25] corroborated clinical evidence of asymmetric and elliptical deployment of the stent frame with both the Evolut PRO and CoreValve devices using an ideal aortic model with BAV. They also demonstrated that the relative position between native leaflets and THV reduces the risk of PVL as assessed by computational flow dynamic. A similar study showed optimal conformability to the elliptical BAV anatomy with both the Lotus and ACURATE THVs [26]. In a single center study focused on n.7 bicuspid patients, Brouwer et al. [27] demonstrated the TAVI outcome with the self-expanding Evolut Pro THV and suggested that simulations can improve the procedural outcome in the challenging bicuspid population. In our previous investigation [28], we assessed the efficacy and safety of TAVI in a cohort of n.8 patients with stenotic BAV who underwent TAVI with the balloon-expandable SAPIEN 3 Ultra device. A detailed analysis and comparison with post-TAVI imaging data highlighted insights on the stent frame eccentricity, valve expansion and risk of PVL. Similarly, Dowling and colleagues [29] evinced that patient-specific computer simulations of TAVI in BAV can be used to identify those patients with the likelihood of unfavorable clinical outcomes. Our findings on the oval expansion of the self-expanding Evolut PRO device and the observation of the regions at highest risk of PVL were in agreement with those reported by the aforementioned studies. However, we recognize a lack of both post-TAVI CT imaging data for validating the deformed THV configuration and functional echocardiography for comparing predicted PVL regions with ultrasound data. Nevertheless, the computer framework here proposed revealed that TAVI in BAV should be evaluated case by case to account for the spatial position and volume of calcifications, with high risk of PVL near the leaflet commissures. In this way, because of limited application of TAVI in BAV, multicenter studies are necessary to confirm the performance of computer simulations in predicting the efficacy of TAVI when deployed in bicuspid patients.

This study has several numerical limitations without showing a comparison with clinical findings to support numerical results. Homogenous isotropic material properties were assumed for both the aortic root and native valve leaflets according to published data [12]. However, the ascending aorta is a heterogeneous and anisotropic material, with diverse biomechanical descriptors from the sinus to the proximal ascending aorta. Although an isotropic model was here adopted, TAVI patients are elderly with stiffening of the aortic wall and changes in elastic fiber architecture leading to isotropic behavior under pathological conditions. However, the modeling with an anisotropic model will likely differently impart the device-related post-TAVI solicitation exerted on the root tissue so that anisotropic modeling should be considered. Simulations have not included the skirt during the implantation because the soft behavior of the sealing skirt likely has low impact on the deployed device configuration. Further developments will be carried out to investigate the impact of the skirt and the constitutive modeling differences on the simulation outcome. Similarly, the calcification plaques are known to have a wide range of stiffness and were simulated in this study as a volume of stiff material that interacts with BAV leaflets. Other research groups [21,25] modeled the stiff plaque inside the leaflet tissue wall. Thus, the impact of different simulation approaches on numerical performance needs to be investigated. Aortic wall pre-stress was also neglected due to the lack of blood pressure data. These numerical assumptions suggest that results need a cautious interpretation, although these appear reasonable in light of the assumptions here adopted. This study can be considered a first step for developing the computational methodology of TAVI in BAVs to confirm the potential role of numerical simulations for supporting the pre-operative planning in this complex patient population. Most importantly, findings were not validated by post-TAVI imaging (using, for example, ultrasounds and CT). This study aimed to develop a framework to measure the deployment of THV in the bicuspid population and estimate several clinically relevant parameters such as the contact pressure and flow velocity. A validation strategy based on post-TAVI CT data to measure the asymmetric THV expansion and functional echocardiography to quantify PVL is already planned for the near future. At the same time, innovative computational technologies as those shown by the Living Heart Human model will be investigated to test the THV performance in a realistic and high-fidelity heart model.

## 5. Conclusions

Recent studies have demonstrated that computational simulations can represent a powerful tool for the virtual planning of TAVI but few of them are focused on bicuspid patients. In this study, a computational framework for the analysis of the structural and hemodynamic performance of THV in patients with severe BAV stenosis is proposed. The THV stent frame was found to expand asymmetrically with differences from patient to patient. The contact pressure and flow velocity map were investigated to visualize the anchoring regions between the inner aortic wall and the THV surface and the region at highest risk of PVL. This study represents a further step towards the assessment of the efficacy and safety of TAVI in bicuspid patients.

## Figures and Tables

**Table 1:** Patient demographics and pre-TAVI CT data.

Age, years	76.2 ± 12.4
Male, %	80
BAV Phenotype	
Pure	2
Left-Right Cusp Fusion	4
Pre-operative CT imaging	
Annulus Area, mm^2^	416.4 ± 102.3
Mean Annulus Diameter, mm	22.5 ± 2.4

## Supplementary Materials

The following are available online at https://www.mdpi.com/article/10.3390/bioengineering8070091/s1, Video S1: Stent deployment.
